# Antifebrin, a New Antipyretic

**Published:** 1886-12

**Authors:** 


ANTIFEBRIN, A NEW ANTIPYRETIC.—Cohn and Heppe have discovered a new antipyretic which promises to revolutionize antifebrile medication. Hitherto all antipyretics belonged either to the phenol series, (carbolic acid, resorsin, salicylic acid, etc.,) or to bases analogous to quinine, (chinolin, chairin, antipyrin, thalline.) The authors have found a substance neither acid nor basic, having the formula, $C^6 H^5 N H C^2 H^3 O$, possessing a powerful antipyretic action, hence its name, *antifebrin*. Antifebrin is a white, crystalline, inodorous powder, with an acrid taste, and insoluble in alcohol. The drug is well borne by the stomach, the quantity employed varying with the severity and stage of the disease, from half a gram to two grams in twenty-four hours. It is most conveniently prescribed in twenty-five centigram doses in warm water or wafers.

Antifebrin is four times more powerful than antipyrin, since with twenty-five centigrams of the former the same effect may be obtained as with one gram of the later. The antipyretic action usually begins one hour after the administration, reaching its maximum effect in four hours, and lasts three hours or more. After each dose the temperature becomes normal, or even descends below normal. At the same time the pulse is reduced in frequency and the arterial tension is increased. During the period of apyrexia there is a return of appetite, thirst and an increased secretion of urine. No one of the twenty-four patients on whom the drug was used exhibited any untoward symptom whatever. On the contrary all declared that they felt much better while under its influence. A few patients, however, were affected with a slight cyanosis of the extremities.

